# Orange Carotenoid Protein in Mesoporous Silica: A New System towards the Development of Colorimetric and Fluorescent Sensors for pH and Temperature

**DOI:** 10.3390/mi14101871

**Published:** 2023-09-29

**Authors:** Silvia Leccese, Andrea Calcinoni, Adjélé Wilson, Diana Kirilovsky, Donatella Carbonera, Thomas Onfroy, Claude Jolivalt, Alberto Mezzetti

**Affiliations:** 1 Sorbonne Université, CNRS, Laboratoire de Réactivité de Surface (LRS), 4 Place Jussieu, 75005 Paris, Franceandrea.calcinoni@studenti.unipd.it (A.C.); claude.jolivalt@upmc.fr (C.J.); 2 Department of Chemical Sciences, University of Padova, 35131 Padova, Italy; donatella.carbonera@unipd.it; 3 Institute for Integrative Biology of the Cell (I2BC), CEA, CNRS, Université Paris-Saclay, CEDEX, 91198 Gif-sur-Yvette, Francediana.kirilovsky@cea.fr (D.K.)

**Keywords:** carotenoids, mesoporous silica nanoparticles, optical sensors

## Abstract

Orange carotenoid protein (OCP) is a photochromic carotenoprotein involved in the photoprotection of cyanobacteria. It is activated by blue-green light to a red form OCP^R^ capable of dissipating the excess of energy of the cyanobacterial photosynthetic light-harvesting systems. Activation to OCP^R^ can also be achieved in the dark. In the present work, activation by pH changes of two different OCPs—containing echinenone or canthaxanthin as carotenoids—is investigated in different conditions. A particular emphasis is put on OCP encapsulated in SBA-15 mesoporous silica nanoparticles. It is known that in these hybrid systems, under appropriate conditions, OCP remains photoactive. Here, we show that when immobilised in SBA-15, the OCP visible spectrum is sensitive to pH changes, but such a colorimetric response is very different from the one observed for OCP in solution. In both cases (SBA-15 matrices and solutions), pH-induced colour changes are related either by orange-to-red OCP activation, or by carotenoid loss from the denatured protein. Of particular interest is the response of OCP in SBA-15 matrices, where a sudden change in the Vis absorption spectrum and in colour is observed for pH changing from 2 to 3 (in the case of canthaxanthin-binding OCP in SBA-15: λ_MAX_ shifts from 454 to 508 nm) and for pH changing from 3 to 4 (in the case of echinenone-binding OCP in SBA-15: λ_MAX_ shifts from 445 to 505 nm). The effect of temperature on OCP absorption spectrum and colour (in SBA-15 matrices) has also been investigated and found to be highly dependent on the properties of the used mesoporous silica matrix. Finally, we also show that simultaneous encapsulation in selected surface-functionalised SBA-15 nanoparticles of appropriate fluorophores makes it possible to develop OCP-based pH-sensitive fluorescent systems. This work therefore represents a proof of principle that OCP immobilised in mesoporous silica is a promising system in the development of colorimetric and fluorometric pH and temperature sensors.

## 1. Introduction

Orange carotenoid protein (OCP) is a water-soluble light-sensitive protein involved in the photoprotection of cyanobacteria [1,2]. In the resting form of OCP, a keto-carotenoid (3-hydroxyechinenone, echinenone (ECN) or canthaxanthin (CAN)) is embedded—without being covalently linked—between two structural domains of OCP (called N-terminal domain—NTD and C-terminal domain—CTD). Absorption of blue-green light triggers large-scale structural modifications of OCP, so that OCP changes from the resting, dark-adapted orange state (OCP^O^), to its active red state (OCP^R^). Only OCP^R^ can induce the quenching of absorbed energy, preventing photo-damage of the photosynthetic apparatus. Specific interactions between amino acid side chains and carotenoid moieties, as well as between specific amino acids, are believed to play a key role in the photoactivation mechanism [3,4,5]. The activation process has been demonstrated to entail deep changes in the structure and shape of the protein [6,7].

OCP activation can be also chemically induced, for instance, by thiocyanate [8], by Cu+ [9] or upon binding to specific silica surfaces [10].

Recently, OCP has been employed for applicative purposes, ranging from a photoprotective element in artificial energy transfer assemblies [11] to a photoactive element of composite photochromic materials [10]. In addition, OCP-fluorescent protein chimeras have been used as temperature sensors [12], whereas OCP has been proposed as an optical sensor to monitor the defrosting of food [13]. Possible applications in synthetic biology and optogenetics have also been proposed for OCP and related proteins, relying on the particular modular NTD–CTD structure of OCP [14].

Structured mesoporous materials are considered of great interest for their particular properties such as a highly reproducible structure with narrow pore size distribution, wide pore diameter range, high surface area and pore volume [15,16]. Thanks to these structural and textural particularities, they are widely considered for applications involving proteins and enzymes, as they are solid supports which are likely to preserve the stability of these biomolecules as well as their mechanism of action [17]. In fact, the immobilisation of proteins—and the putative stabilisation of their native conformation after the interaction with mesostructured surfaces—is the object of intense research in a wide range of nanobiotechnological applications ranging from biocatalysis to sensors, or even tissue engineering or nanomedicine [16,17,18,19,20].

Among the mesoporous materials able to immobilise proteins [21], SBA-15 (Santa Barbara Amorphous) has attracted much attention as a host for immobilisation of enzymes as well as delivery systems [21]. SBA-15 was synthesised for the first time in 1998 [22] and is an ordered mesoporous silica material with regular, hexagonal pores 200 nm up to a few μm long. A key point is that the size of these pores can be tuned from 2 to 50 nm wide. Smaller pores connect mesopores with each other [23]. Furthermore, SBA-15 is characterised by uniform porosity, thermal stability, good mechanical stiffness, a high pore volume and a large specific surface area. The structural parameters of SBA-15 (e.g., the diameter of pores) can be adjusted by appropriately tuning the parameters used during its synthesis [24]. The possibility to modify the surface properties of these materials can provide higher protein immobilisation capacity and better preservation of its stability and activity [21,25]. Finally, SBA-15 is non-toxic.

In a recent work [10], we have immobilised two different kinds of OCP (binding either ECN or CAN) on different kinds of raw and surface-functionalised (by amino-propyl groups) SBA-15 mesoporous silica nanoparticles. SBA-15 matrices have been demonstrated to be suitable supports for both OCPs, which remain photoactive inside the mesoporous matrix, thereby producing photochromic nanoparticles.

**Figure 1:**
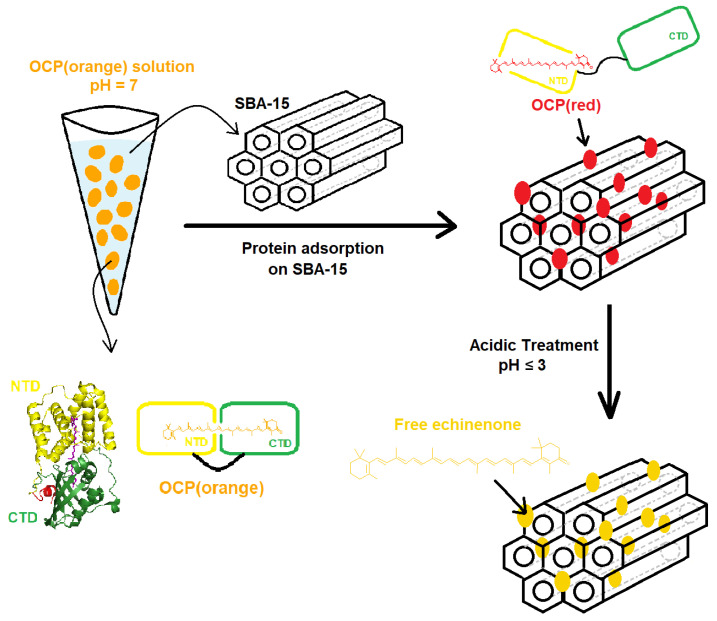
Scheme for the encapsulation of OCP(ECN) in SBA-15 mesoporous silica nanoparticles and the response of the developed hybrid system to acidic pH.

Here, we present new data on the behaviour, in the dark, of OCP binding either ECN or CAN, in particular when encapsulated in SBA-15 nanoparticles. First, we demonstrate that, both in its free and SBA-15-bound forms, OCP can change its colour (because of chemical activation or because of protein denaturation) at particular pH values. In the case of non-surface-functionalised SBA-15 matrices, the effect of the pH is considerably different compared to the one we observed in solution (notably, at acidic pH; see Figure 1).. We also show that specific fluorophores can be encapsulated along with OCP in the same SBA-15 mesoporous nanoparticle (whose surface is functionalised by aminopropyl groups) and that in the resulting systems, the pH-induced activation of OCP at acidic pH can induce a quenching of fluorescence. This represents a key test to show that the development of a pH-sensitive “turn-off” fluorescent nanodevice based on OCP is possible.

This work therefore represents a proof of principle that OCP in SBA-15 can be a suitable system for the development of colorimetric and fluorometric pH sensors in acidic media. It should be mentioned that pH sensors based on pH-sensitive proteins which change their electronic spectra encapsulated in porous supports have already been reported and used to investigate the effective pH inside pores [26].

The work has been completed by an investigation into the effect of temperature on OCP encapsulated in SBA-15 matrices. Interestingly, the temperature-induced activation and denaturation processes were found to depend on the type of SBA-15 matrix used, as well as on the carotenoid (CAN or ECN) bound to OCP. Such an investigation gives two important pieces of information: first, it clearly shows that the environment surrounding OCP deeply influences its stability (folded or denatured) and its state (activated—red, or non-activated—orange). The chemical structure of the carotenoid (CAN has two C=O groups, ECN only one) also has a strong effect on the temperature response of the nanosystems. Second, it again represents a proof of principle that OCP in SBA-15 can be a suitable system for the development of colorimetric sensors for temperature changes.

## 2. Materials and Methods

### 2.1. Expression of Synechocystis-OCP in E. coli

Recombinant OCP(ECN) and OCP(CAN) from *Synechocystis* PCC 6803 were produced in *E. coli* following the protocol reported by Bourcier de Carbon et al. [27].

### 2.2. Synthesis of SBA-15

SBA-15 was synthesised as reported in the literature using a triblock copolymer, Pluronic P123 ((EO)20(PO)70(EO)20 from Aldrich, Darmstadt, Germany) as a structure-directing agent. In a typical synthesis, 8.02 g of P123 was mixed under stirring (at 750 rpm) with 280 mL of 0.1 M HCl for 2 h at 40 °C until the complete dissolution of P123 and the creation of a mesostructured phase of micelles. Then, 16.80 g of tetraethyl-orthosilicate (TEOS; Aldrich; 98%) as silica precursor was added—in a drop-by-drop procedure—to the solution under slower stirring (at 500 rpm). The resulting suspension was left under stirring for 24 h to obtain the formation of a mesostructured silica sol–gel. The sol–gel suspension was then transferred into a 1 L glass bottle and hydrothermally treated for 24 h at 80 °C or 120 °C. Finally, the obtained solid was filtrated and thoroughly washed with approximately 4 L of distilled water to get rid of the P123 polymer. The resulting powder was dried at 80 °C or 120 °C overnight and calcined at 550 °C (heating ramp: 0.4 °C min^−1^) for 6 h under air to remove the template. Finally, roughly 4 g of SBA-15 material was obtained. By the appropriate setting of experimental conditions, in particular the hydrothermal step nanoparticles with different pores, diameters were obtained. In particular, nanoparticles with a pore diameter of 6 nm were obtained after the 24-h hydrothermal treatment at 80 °C; hereafter, these nanoparticles will be called SBA-15-80. Nanoparticles with pore diameter of 8 nm were obtained after the 24-h hydrothermal treatment at 120 °C; hereafter, these nanoparticles will be called SBA-15-120.

### 2.3. Surface Functionalisation: SBA-15-NH_2_

Functionalisation of the SBA-15 surface by aminopropyl groups was performed as reported in Leccese et al. 2022 [10]. In short, prior to the functionalisation, 1g of SBA-15 was placed in a reactor and activated at 350 °C (heating ramp: 5 °C min^−1^) for 3 h under 50 mL min^−1^ air flow. The sample was then transferred into a dried balloon equipped with a septum. Subsequently, 50 mL of commercial anhydrous toluene (VWR, Fontenay sous Bois, France; 99.9%) was added to silica. The balloon was then flushed with argon for 10 min and then left for 5 min in an ultrasonic bath to obtain a perfect silica suspension. The balloon was finally connected to a refrigerator and placed under argon flow. To start the functionalisation reaction, 1 mL of 3-aminopropyltriethoxysilane (ATPES; Aldrich; 99%) was added to the suspension drop by drop. Then, the argon flow was stopped, and the suspension was stirred for 1 h at 25 °C and then for 24 h refluxing (120 °C). Finally, the mixture was filtrated on Büchner and filter paper and washed with 30 mL of anhydrous toluene (VWR; 99.9%), 30 mL of acetonitrile (VWR; HPLC grade) and 30 mL of absolute ethanol (Aldrich; 99.9%). The resulting solid was dried at 60 °C overnight. The excess of APTES was extracted with a Soxhlet apparatus, introducing 50 mL of CH_2_Cl_2_ (Carlo Erba Reagents, Dasit Group SpA, Milan, Italy; 99.9%) in a balloon and refluxing the system at 45 °C for 24 h. Finally, the functionalised silica was dried at 60 °C overnight.

These surface-functionalised nanoparticles are hereafter named SBA-15-NH_2_ (more precisely: SBA-15-80-NH_2_ for those deriving from SBA-15-80; SBA-15-120-NH_2_ for those deriving from SBA-15-120).

### 2.4. Electronic Microscopy

The morphology of the nanoparticles was characterised with a high-resolution SEM-FEG Hitachi SU-70 operating at an acceleration voltage of 1.0 kV, and at a working distance of 3 mm. Transmission electron microscopy (HRTEM) was carried out on a JEOL JEM 1011 (W) microscope operating at 100 kV and equipped with an ORIUS Gatan Camera. For TEM observations, the sample powders were deposited on a 3 mm copper grid coated with an amorphous carbon film. Samples were prepared by dispersing the nanoparticles in ethanol using ultrasonic cleaner. A drop of the suspension was then put on carbon films on copper grids.

### 2.5. Immobilisation of OCP on SBA-15 and on SBA-15-NH_2_

A total of 1 mL of a 0.2 mg/mL OCP solution was mixed with 10 mg silica powder. The mixture (pH = 8.5) was incubated under gentle stirring for 4 h at 298 K in the dark, to avoid the photo-activation of the protein, then overnight at 4 °C. More details can be found in Leccese et al. 2022 [10].

### 2.6. Immobilisation of Fluorophores Cy3 and Cy5 on SBA-15-NH_2_

Experiments were carried out using 4 μM stock solutions of cyanine dyes Cy3 and Cy5 (Lumiprobe) in Milli-Q water (pH 7 for Cy3 and pH 6.5 for Cy5). In order to obtain a 1:9 Cy3:Cy5 ratio, 0.1 mL of Cy3 stock solution was added to 0.9 mL of Cy5 stock solution. This 1:9 ratio was selected to maximise the detectable emission from Cy5 [25]. The overall 1.0 mL mixed solution was put in contact with 10 mg of SBA-15-80-NH2 powder and gently stirred for 60 min. After centrifugation, Cy3/Cy5-loaded SBA-15-80-NH_2_ nanoparticles were collected and analysed with UV-Vis and fluorescence spectroscopy.

To carry out the subsequent immobilisation of OCP on Cy3/Cy5-loaded SBA-15-80-NH_2_ nanoparticles, 1 mL of OCP(CAN) 0.2 mg/mL was added to the dye-loaded SBA-15-80-NH_2_ pellet and gently stirred for 4 h on a moving plate, in the dark, to avoid the photo-activation of the protein. Then, the sample was centrifuged for 30 min at 8000 rpm, and the OCP/Cy3/Cy5 SBA-15-80-NH_2_ analysed with UV-Vis and fluorescence spectroscopy.

### 2.7. UV-Visible Spectroscopy in Liquid and Solid Phases

UV-Vis absorption spectra of OCP solutions were recorded on a Biochrom Libra S60 spectrophotometer, between 350 and 750 nm with a resolution of 2 nm.

UV-Vis absorption spectra of solids (OCP encapsulated in SBA-15) were recorded on a Varian 2300 UV-Vis spectrophotometer equipped with an integrating sphere between 350 and 750 nm with a resolution of 1 nm and an acquisition time of 0.1 s per point.

### 2.8. Fluorescence Spectroscopy

Fluorescence was measured on a Horiba Scientific Fluorolog 3 apparatus. The instrument is composed of a xenon lamp at 450 W, equipped with a dual array monochromator (Grating 1200 gr/mm) to select the incident wavelength, with a dual array monochromator (Grating 1200 gr/mm) to analyse the emission and with a photomultiplier-type detector (PM R928).

## 3. Results

### 3.1. Effect of pH on OCP Vis Spectra

**Figure 2:**
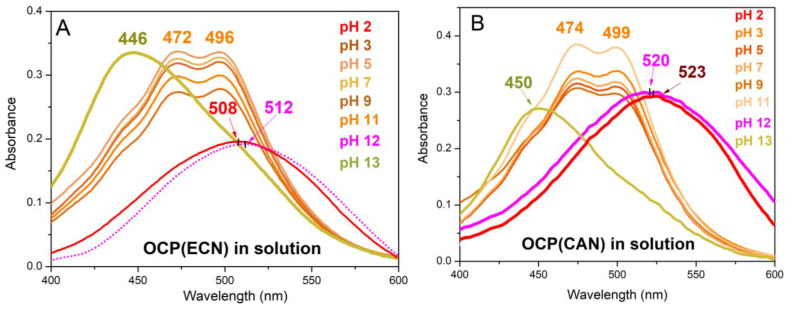
(**A**) Vis absorption spectrum of OCP(ECN) in solution at different pH levels. (**B**) Vis absorption spectrum of OCP(CAN) in solution at different pH levels.

In Figure 2, visible absorption spectra recorded on OCP(ECN) and OCP(CAN) in solution at different levels of pH are shown.

As it can be observed, at a pH between 3 and 11, the visible absorption spectra are typical of the resting orange form of OCP, with peaks at 472–474 and 496–499 nm, due to the vibronic structure. At a very acidic pH (pH = 2) for both OCP(CAN) and OCP(ECN), an evident red-shift is observed (508 nm for OCP(ECN); 523 nm for OCP(CAN)). Conversely, at a basic pH, the situation is quite complicated. Whereas for 3 ≤ pH ≤ 11, the shape of the Vis absorption spectrum is typical of the orange form, at pH = 12, a clear indication of the formation of the red form is observed (λ^max^_abs_ in the 510–520 nm range for both OCPs). However, at pH = 13, the Vis spectrum resembles that of the carotenoid in solution [28,29], showing peaks at 446 nm (ECN) or 450 nm (CAN). We take this evidence as an indication of the denaturation of the protein.

This suggests that both OCP(ECN) and OCP(CAN) in solution can be considered optical pH sensors, capable of a two-colour transition at an acidic pH (pH = 2: red; pH = 3: orange) and of a three-colour transition at a basic pH (pH = 11: orange; pH = 12: red; pH = 13: yellow).

**Figure 3:**
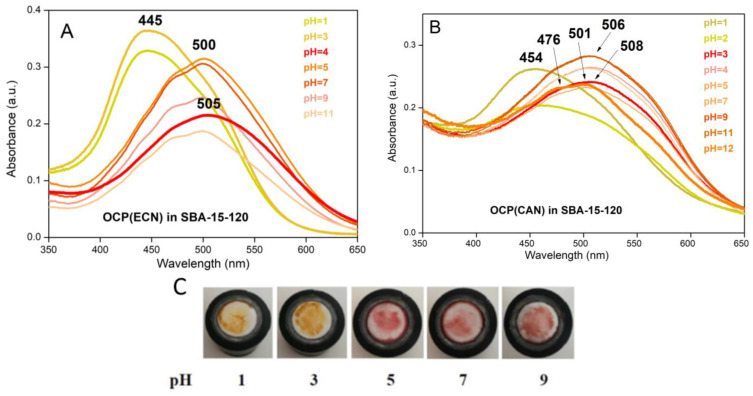
(**A**) Vis absorption spectrum of OCP(ECN) in SBA-15-120 silica mesoporous nanoparticles at different pH levels. Experiments were carried out as follows: 10 mg of OCP-loaded silica nanoparticles was added to 1 mL of solution at a given pH. The obtained suspension was stirred for 30 min. After centrifugation, the obtained pellet was analysed using UV-Vis spectroscopy. (**B**) Vis absorption spectrum of OCP(CAN) in SBA-15-120 silica mesoporous nanoparticles at different pH levels. Experiments were carried out as in (**A**). (**C**) Pictures of sample-holder of samples of OCP(ECN) in SBA-15-120 treated at different pH levels: 1, 3, 5, 7 and 9 (left to right).

Figure 3 shows the pH dependence of OCP(ECN) and OCP(CAN) encapsulated in SBA-15-120 mesoporous silica nanoparticles (SEM and TEM images of the samples are shown in Figure S1 of the ESI). Very interestingly, the situation at acidic pH is reversed in both systems compared to the situation found for the two proteins in solution. For OCP(ECN)@SBA-15-120, for 1 ≤ pH ≤ 3, the system is characterised by a 445 nm band characteristic of free ECN [28] (Figure 3A), and it appears yellow (Figure 3C). At pH = 4, the situation changes abruptly, with a sudden 60 nm redshift to 505 nm (Figure 3A); the system now looks red. At higher pH levels (5–11), the colour slightly shifts more towards orange (Figure 3C), with a maximum absorption peak at 500 nm and a shoulder at ~477 nm. At more basic pH levels, the absorption band becomes very broad, indicative of the simultaneous presence of several species.

The situation is even more interesting in the case of OCP(CAN)@SBA-15-120. At pH = 1, the system is yellow, with an absorption band of free canthaxanthin at 454 nm [24] (Figure 3B). The situation changes completely at pH = 3: the system becomes red (λ^max^_abs_ = 508 nm) with a 54 nm redshift (Figure 3B). At pH = 2, a kind of intermediate situation is found (λ^max^_abs_ = 455 nm but presence of a significant shoulder at ~505 nm). For 3 < pH ≤ 11, the situation is essentially equivalent to pH = 3 (λ^max^_abs_ = 505 ÷ 506 nm). At pH ≥ 12, the system becomes more orange (λ^max^_abs_ = 501 nm with a strong shoulder—nearly a twin peak—at 476 nm).

However, it should be kept in mind that SBA-15 mesoporous matrices are known to undergo partial dissolution at pH > 7 [30]. Therefore, the response of OCP in SBA-15 at a basic pH should be interpreted with extreme care.

To summarise, the most evident feature for both systems is the sudden spectral and colour changes (54–60 nm shift in λ^max^_abs_) upon slight variation of the pH in acidic conditions. It is interesting to note that the situation is nearly opposite (in colours) to what is observed in solution, where the red form of the protein is observed for pH ≤ 2. It should be noted that SBA-15 were reported to be unstable only under extremely acidic conditions [31]. We are therefore confident that in the pH range explored in this work, the colour change upon pH changes comes directly from the protein response. OCPs in SBA-15-120 nanoparticles are therefore in principle exploitable as colorimetric nanosensors in the acidic pH range (1–7). Due to the size of the SBA-15 particles (about 0.2–0.4 µm width and 1–2 µm length), it would be possible to probe pH variation at a micrometric scale. As an example, our system could be then used in a microfluidic set-up or in confined media to detect local pH changes, for instance, within a single SBA-15 particle.

### 3.2. OCP-Mediated Effect of pH on Cy3-Cy5 Fluorescence

**Figure 4:**
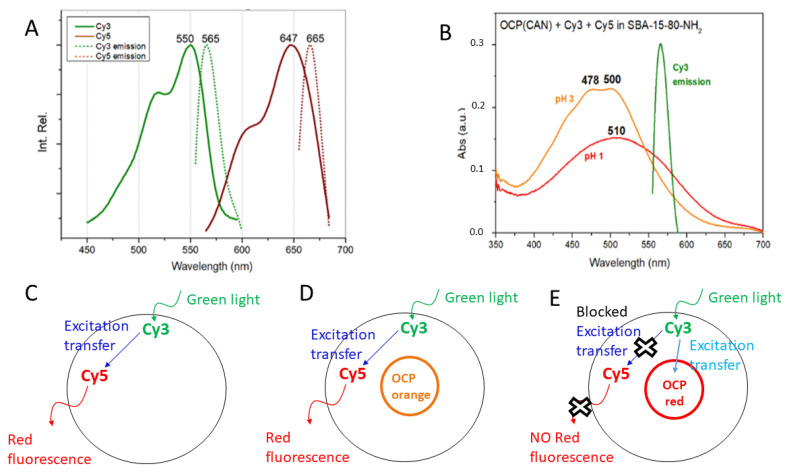
(**A**) Absorption and emission spectra of Cy3 and Cy5 in SBA-15-NH_2_ nanoparticles. (**B**) Absorption spectra of OCP(CAN) in SBA-15-NH_2_ nanoparticles at pH = 3 and pH = 1. The emission spectrum of Cy3 in SBA-15-NH_2_ is also shown, to underline its spectral overlap with OCP absorption spectra. (**C**) Example of Cy3-Cy5-loaded SBA-15-NH_2_ nanoparticles: absorption at 550 nm by Cy3 is followed by excitation transfer to Cy5 and then by Cy5 emission at 665 nm. (**D**) Example of Cy3-Cy5-OCP-loaded SBA-15-NH_2_ nanoparticles at pH = 3 (condition under which OCP is orange): absorption at 550 nm by Cy3 is followed by excitation transfer to Cy5 (excitation transfer to OCP^O^ is negligible), and Cy5 emission at 665 nm is observed. (**E**) Example of Cy3-Cy5-OCP-loaded SBA-15-NH_2_ nanoparticles at pH = 1 (condition under which OCP is red): absorption at 550 nm by Cy3 is followed by excitation transfer to OCP^R^, and Cy5 emission at 665 nm is strongly diminished compared to pH = 3.

Following the approach of Andreoni et al. [11], we developed a system in which OCP could act as a switch in blocking the energy transfer between two cyanine dyes, Cy3 and Cy5. The rationale is that in solution, the emission spectrum of Cy3 overlaps partially both with absorption spectra of Cy5 and of OCP^R^, while the overlap with the absorption spectrum of OCP^O^ is much less. The same holds true for Cy3, Cy5 and OCP^R^ and OCP^O^ in SBA-NH_2_ matrices (see Figure 4A,B). We used OCP(CAN) in SBA-15-80-NH_2_ mesoporous nanoparticles (for SEM and TEM images of these nanoparticles, see [10]) which has a pH response between 1 and 7 similar to the one obtained for OCP(CAN) in solution, with OCP^R^ formed at pH 1 (see Figure S2 in ESI for Vis spectra of OCP(CAN) at 1 ≤ pH ≤ 7).

Details on the molecular architecture of the system and on the effect of OCP photoactivation by blue light are the object of a forthcoming manuscript (Leccese et al., in preparation) but can also be found in [32]). Briefly, Cy3 and Cy5 are co-adsorbed in SBA-15-NH_2_ nanoparticles. In the absence of OCP, light at 550 nm is absorbed by Cy3. Through an energy-transfer mechanism, or (more likely) an emission-absorption mechanism (Cy3 fluoresces at 565 nm, where Cy5 absorbs), excitation is transferred to Cy5 (see Figure 4C). Finally, fluorescence from Cy5 is observed at 665 nm. Let us now consider the system where OCP in its orange state is adsorbed in SBA-15-NH_2_ simultaneously with Cy3 and Cy5: the Vis absorption spectrum of OCP^O^ does not overlap significantly with the Cy3 fluorescence peak. As a consequence, Cy3 absorbs light at 550 nm; then, an excitation transfer from Cy3 to Cy5 can take place (see Figure 4D), and 665 nm Cy5 fluorescence is observed. Conversely, when OCP is turned into the red form (in the present case, this is induced by a pH decrease), its Vis absorption spectrum superimposes largely over the Cy3 emission. Therefore, excitation at 550 nm of Cy3 is followed by excitation transfer from Cy3 to OCP^R^, and not to Cy5. As a result, Cy5 fluorescence is quenched (Figure 4E).

**Figure 5:**
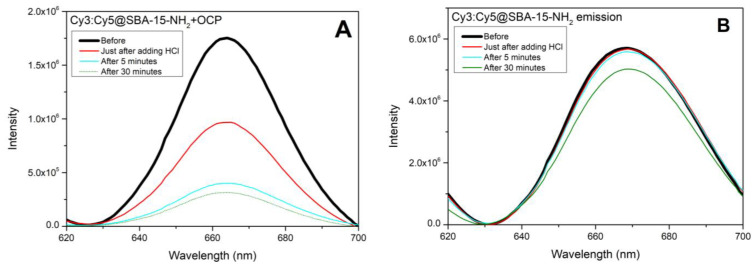
(**A**) Emission from Cy3/Cy5/OCP-loaded SBA-15-80-NH_2_ nanoparticles at neutral pH and at different times after addition of a drop of concentrated HCl solution. Before HCl addition, OCP is orange, and upon excitation at 550 nm, the excitation is transferred to Cy5. After adding HCl, fluorescence of Cy5 is progressively quenched as a result of progressive acidic pH-induced red-conversion of OCP and consequent excitation transfer to Cy3 to OCP^R^. (**B**) The same experiment, but in Cy3/Cy5-loaded SBA-15-80-NH_2_ nanoparticles. Fluorescence spectra are recorded at neutral pH and at different times after addition of a drop of concentrated HCl solution. OCP being absent, the decrease in fluorescence in this case is due to mesoporous silica surface modification by the acidic treatment and/or Cy3/Cy5 response to acidic pH. The fluorescence decrease is much smaller than in case (**A**) and only becomes detectable more than 5 min after HCl addition.

As already mentioned, we decided to use surface-functionalised SBA-15-80-NH_2_ mesoporous nanoparticles for the clear advantage that, in these matrices, OCP(CAN) undergoes red-conversion at pH = 1. Furthermore, these nanoparticles could bind both Cy3 and Cy5 dyes better than non-functionalised SBA-15 nanoparticles, without significant alteration of their absorption and emission properties [32]. It should also be noted that OCP(CAN) encapsulated in SBA-15-NH_2_ matrices gives the most red-shifted OCP^R^ absorption band (508–510 nm) among all OCPs encapsulated in SBA-15 matrices [10]. Indeed, acidic pH-induced experiments show that, at pH = 1, OCP(CAN) in SBA-15-80-NH_2_ nanoparticles is red, with a broad absorption band centred at 510 nm and a shoulder at ~550 nm (see Figure 4B; see also Figure S2 ESI). This ensures, considering all the Vis spectra of immobilised OCPs in the red form in mesoporous silica [10], the best possible conditions for the overlap with Cy3 emission. In order to explore if the fluorescence from Cy5 is quenched when an acidic pH converts OCP to its red form, the following procedure was applied. A fluorescence spectrum (λ_exc_ = 550 nm) was recorded for Cy3/Cy5/OCP SBA-NH_2_ nanoparticles. An intense peak at 665 nm was observed (see Figure 5A). Then, a drop (30 µL) of concentrated HCl solution was added in situ, and a fluorescence spectrum (λ_exc_ = 550 nm) was recorded immediately afterwards. This spectrum showed a peak at 665 nm whose intensity was nearly half compared to the spectrum recorded before HCl addition. Fluorescence spectra were also recorded 5 and 30 min afterward: interestingly, the 665 nm fluorescence peak further decreased (see Figure 5A). We interpreted these data as a consequence of the progressive conversion of OCP from the orange to the red form. OCP^R^ can block excitation transfer from Cy3 and Cy5 and quench the recorded Cy5 fluorescence. This kind of “temporal evolution” in the quenching (and therefore in the pH-induced OCP conversion to the red form) is not surprising, as it has been shown that in SBA-15-80-NH_2_ nanoparticles, OCP(CAN) back-conversion from the red form to the orange form is severely slowed down compared to solution [10]. Therefore, this slowed kinetics (both in pH-induced orange-to-red activation and in back-conversion of the red form to the orange form) may be considered as an effect of the SBA-15-80-NH_2_ matrix on the kinetics of OCP(CAN) activation/back-conversion.

We repeated the same experiment on Cy3/Cy5 SBA-15-80-NH_2_ nanoparticles. In the absence of OCP, any pH-induced modification in the fluorescence spectrum is due to a change in the fluorescence properties of the two dyes and cannot be related to an OCP orange-to-red conversion. In Figure 5B, a series of fluorescence spectra (before, soon after, 5 min after, 30 min after adding HCl) is shown. It is clear that the magnitude of the fluorescence quenching is much less pronounced compared to Cy3/Cy5/OCP SBA-15-NH_2_ nanoparticles and is most likely due to a chemical modification/degradation of the two dyes, or to a modification of the surrounding molecular environment, notably the aminopropyl functionalising groups. Furthermore, it cannot be excluded that the fluorescence quenching reflects a partial chemical modification of the SBA-15 structure, as observed under very acidic conditions [31].

Even the temporal evolution of the fluorescence decrease in HCl-treated Cy3/Cy5 SBA-15-80-NH_2_ nanoparticles follows a different trend compared to HCl-treated Cy3/Cy5/OCP SBA-15-80-NH_2_ nanoparticles. This supports the conclusion that the fluorescence quenching in Cy3/Cy5/OCP SBA-15-80-NH_2_ nanoparticles is mainly due to the pH-induced conversion of OCP^O^ to OCP^R^. In other words, this represents a proof of principle of a possible use of OCP as a pH-sensitive element to develop a nano-sized fluorescent device for detection of acidic pH conditions.

### 3.3. Effect of Temperature on OCP Vis Spectra

This study was completed by investigating the effect of temperature on OCP encapsulated in SBA-15 matrices. We were pushed to investigate this particular aspect by the fact that, in the literature, the use of OCP as a temperature-sensitive element in a bioengineered temperature sensor was reported [12]. Furthermore, a patent based on temperature effects on OCP colour has also been filed [13].

**Figure 6:**
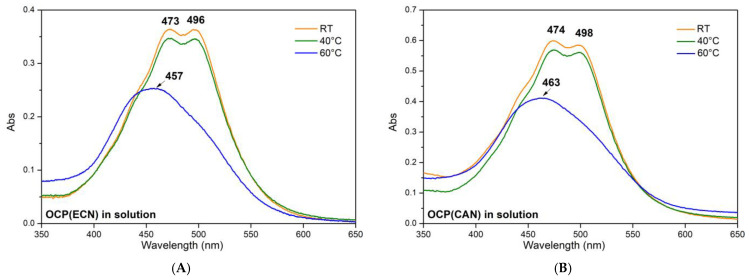
(**A**) OCP(ECN) in solution. (**B**) OCP(CAN) in solution. Absorption visible spectra in the 350–700 nm range. Orange traces: spectra at RT (25 °C). Green traces: spectra after thermal treatment at 40 °C. Blue traces: spectra after thermal treatment at 60 °C. Experiments were carried out as follows: the solution was put in an Eppendorf tube (wrapped in aluminium foil to avoid photo-activation) and then was heated to the desired temperature using a thermostatic water bath for 30 min. Then, the solution was transferred to a cuvette, and a Vis spectrum was recorded within 2 min.

Figure 6 shows the effect of thermal treatment at 40 °C and 60 °C on OCP(ECN) and OCP(CAN) in solution. For comparison, the spectrum at room temperature is also provided.

In both cases, heating of the protein to 40 °C had no detectable effect on the Vis spectrum. Conversely, thermal treatment at 60 °C induced a clear modification of the spectra, which show typical features of free carotenoid in solution in both cases (small traces of OCP in the orange form might still be present). This can be easily interpreted as temperature-induced irreversible denaturation of the protein.

**Figure 7:**
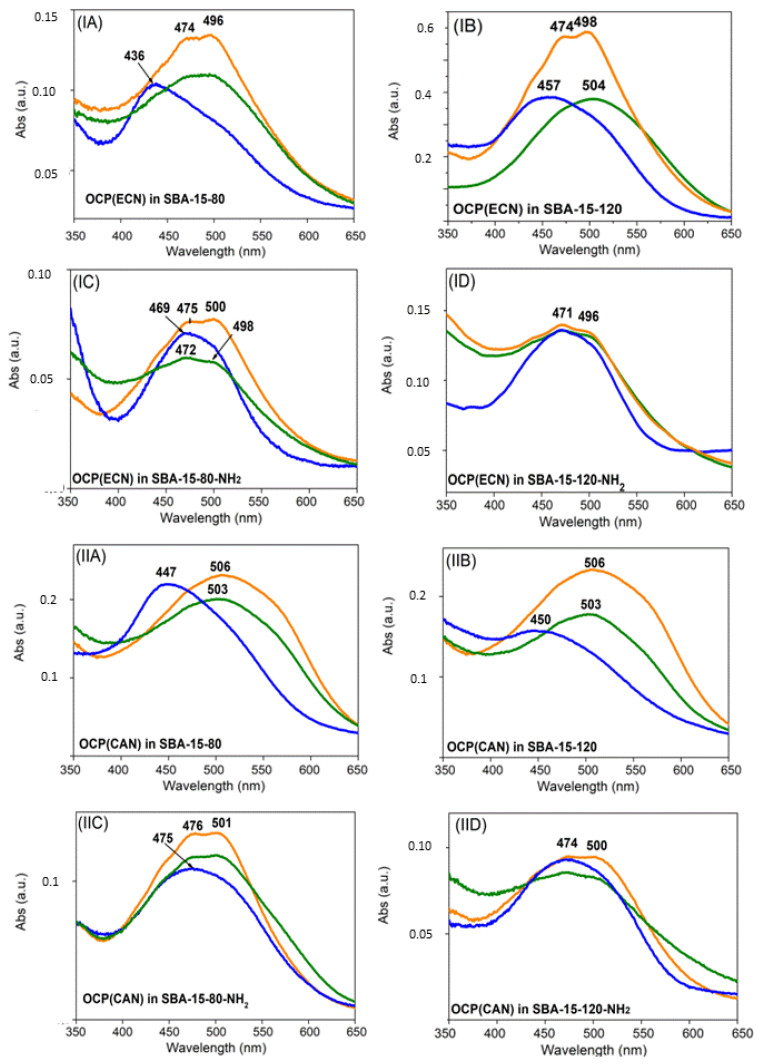
OCP(ECN) in SBA-15-80 (6 nm pore diameter) (**IA**); SBA-15-120 (8 nm pore diameter) (**IB**); SBA-15-80-NH_2_ (**IC**); SBA-15-120-NH_2_ (**ID**); OCP(CAN) in SBA-15-80 (**IIA**); SBA-15-120 (**IIB**); SBA-15-80-NH2 (**IIC**); SBA-15-120-NH2 (**IID**). Absorption visible spectra recorded in 350–700 nm range. (Orange) spectra recorded at room temperature before thermal treatment; (green) spectra of samples treated at 40 °C; (blue) spectra of samples treated at 60 °C. Spectral intensity variability depended on sample preparation before the analysis. Experiments were carried out as follows: the solid sample was heated to the desired temperature using temperature-controlled distilled water in a beaker for 30 min. Then a Vis spectrum of the sample was recorded. The whole procedure was carried out in darkness to avoid photo-activation of OCP.

Figure 7 shows the effect of thermal treatment on the visible spectra of OCP(ECN) and OCP(CAN) in different SBA-15 matrices. Interestingly, the particular kind of SBA-15 matrix seem to play a key role. Two parameters were varied: (1) the pore size (6 nm vs. 8 nm, i.e., SBA-15-80 vs. SBA-15-120); (2) surface functionalisation (raw SBA-15 vs. SBA-15-NH_2_). In addition, OCP(ECN) and OCP(CAN) also show clear differences in their behaviour when inserted into the same SBA-15 matrix.

A particularly interesting situation is found for OCP(ECN) encapsulated in raw (non-surface-functionalised) SBA-15-120 nanoparticles (8 nm pore diameter). While the Vis spectrum at RT shows clear features of the orange form (with possible small contributions from the red form), the situation changes after thermal treatment at 40 °C. The Vis spectrum is now clearly redshifted (λ^max^_abs_ = 504 nm), with loss of the vibronic structure. This can be interpreted as due to a mixture of red form (dominant) with significant contributions from the orange form. The situation changes again after thermal treatment at 60 °C: the maximum absorbance is now at 457 nm, but an important shoulder at ~500 nm is observed. The most likely hypothesis is that this spectrum represents a situation where the majority of the carotenoid is out of the binding pocket in OCP, but a significant contribution from OCP in the red form is also present. From a colorimetric point of view, it can be easily seen that the Vis spectra (and therefore the colours) of the system are different after thermal treatment at the three temperatures, with an interesting sequence (with increasing temperature) of orange (λ^max^_abs_ = 474/497 nm) → orange/red (λ^max^_abs_ = 504 nm) → yellowish (λ^max^_abs_ = 457 nm). In other terms, a three-colour response to temperature is observed.

Also of particular interest is the system composed by OCP(CAN) in raw SBA-80-15 nanoparticles with an average pore diameter of 6 nm. The spectrum is indicative of a red form (with important contributions from the orange form) both at RT (λ^max^_abs_ = 503 nm) and at 40 °C (λ^max^_abs_ = 506 nm), but it undergoes a strong blueshift after thermal treatment at 60 °C (λ^max^_abs_ = 447 nm). Also in this case, small contributions from protein-bound canthaxanthin (most likely in the red form) are present. The ~60 nm shift observed between the spectra recorded after treatment at 40 °C and 60 °C could be of particular interest for a clear colorimetric detection of temperature changes in this range.

Finally, it should be underlined that, in some situations, the mesoporous matrix seems to act—at least partially—as a “preserving agent” against temperature-induced protein denaturation: this is, for instance, the case of OCP(ECN) in SBA-15-120-NH_2_ where, even after treatment at 60 °C, the Vis spectrum almost completely conserves the spectra features of the orange form.

## 4. Discussion

In a previous paper, we studied the photoresponse of OCP bound to mesoporous silica matrices, showing that in SBA-15 mesoporous silica, composite photochromic hybrid nanomaterials could be obtained [7]. Interestingly, OCP(CAN) and OCP(ECN) were found to have different spectral and photochromic behaviours when inserted in SBA-15 (raw and surface-functionalised) nanoparticles. The dimensions of the pores and the chemical modification of the internal surfaces of the mesoporous matrix (i.e., functionalisation by aminopropyl groups) were also found to have key roles.

In the present study, the dependence on pH and temperature of OCP activation in the same nanosystems—as well as in solution—was investigated. Whereas a systematic study of the effect of pH and temperature on OCP was—to the best of our knowledge—lacking (the only papers partially investigating it are [33,34], both focusing on OCP binding 3′-hydroxyechinenone), it is interesting to note that OCP has been previously shown to act as a temperature-sensitive element in bioengineered temperature sensors [12]. Furthermore, several reports from different research groups [8,9,10] had demonstrated that chemical activation of OCP (from the orange to the red form) in the dark is also possible.

Here, we show that OCP (binding both ECN and CAN) in solution can be activated to the red form at acidic pH (pH = 2). For 3 ≤ pH ≤ 11, OCP is orange. Interestingly, at pH = 12, activation to OCP^R^ is observed, whereas at pH = 13, the spectrum of the free ketocarotenoid is recorded, indicating denaturation of the protein. This is in quite good agreement with published data for OCP binding 3′-hydroxyechinenone, for which red forms at pH = 12 [34] and pH = 3.5 [33] have been reported.

Therefore, OCP (binding ECN or CAN) in solution can act as a colorimetric pH sensor both in acidic conditions (red for pH ≤ 2, orange for pH ≥ 3) but also in basic conditions (in this case, with a three-colour behaviour: orange for pH ≤ 11; red at pH = 12; yellow at pH ≥ 13). A very similar behaviour in the acidic range was also observed when the two kinds of OCPs were encapsulated in SBA-15-80-NH_2_ matrices (see Figures S2 and S3 in ESI), which made it possible to develop Cy3/Cy5-based fluorescent on/off sensors for pH ≤ 1.

The results for the raw SBA-15 nanoparticles are also particularly interesting, as they show the most evident colour changes (~60 nm) in selected narrow intervals of pH (for OCP(ECN), a dramatic colour change is observed at acidic pH: yellow at pH ≤ 3, red at pH = 4, red/orange for pH ≥ 5; for OCP(CAN) the behaviour is similar, with the turning point downshifting from pH = 4 to pH = 3).

These pieces of information are also particularly interesting for several other reasons:

(1) They can help us better understand the mechanism of photo-activation, which is under intensive study and the object of lively debate ([3,35,36,37,38,39] and refs. therein);
(2) They provide new information concerning the influence of the surrounding environment on the orange to red activation;
(3) They show that OCP in SBA-15 matrices is a promising system for the development of nano-sized optical sensors for pH. In this framework, the obtained results on the fluorescent Cy3/Cy5/OCP SBA-15-80-NH_2_ nanoparticles show that mesoporous silica can act as a scaffold to build up more elaborate nanodevices based on OCP pH orange-to-red conversion, for the fluorescence detection of pH changes.

In this framework, we show a comparison of pH sensors based on proteins encapsulated in mesoporous silica matrices ([26,40,41] in Table 1, underlining the advantages/drawbacks of the OCP-based hybrid systems presented in this paper.

Also, the response of the OCP/SBA-15 systems to temperature is particularly interesting, showing a significant effect of the matrix. Whereas it is difficult—at the present stage—to explain the observed behaviours for the Vis spectra recorded after thermal treatment, it is quite clear that some systems show an increased thermal stability compared to solution. More interestingly, some other systems—namely OCP(ECN) in raw SBA-15-120 nanoparticles and OCP(CAN) in raw SBA-80-15 nanoparticles—show interesting spectral responses to thermal treatment, making them possible candidates for the development of nano-sized colorimetric temperature sensors. Unfortunately, for the time being, our experimental photoluminescence apparatus does not allow us to carry out fluorescence experiments on thermally treated dye/OCP-loaded SBA-15 systems. We hope these experiments will be possible in the near future.

It is also interesting to point out that the most interesting results for the spectral response to thermal treatment were obtained for raw, non-surface-functionalised OCP-loaded SBA-15 nanoparticles. This is an opposite situation compared to the results obtained when the photochromic response is investigated [10]. In that case, the surface functionalisation of SBA-15 nanoparticles by aminopropyl groups represented a key point to obtaining the most interesting results [10].

The present study therefore represents a starting point: different kinds of photoactive OCPs (from other organisms, or from available mutants) may respond differently to pH (both in solution and bound to mesoporous silica matrices). Indeed, this seems to be the case for OCP binding 3′-hydroxyechinenone in solution, which turns red at pH = 3.5 [33]. Also, OCPs binding different carotenoids (e.g., zeaxanthin; this OCP is not photo-active) can be tested. The investigation can also be extended to OCP-related carotenoproteins (HCP, CTDH) [1,14] because, in the development of pH colorimetric sensors, the main purpose is to detect a clear colour change depending on the pH, even it is not related to the orange-to-red photoconversion of OCP. The key point is to induce a change of the colour of the carotenoid (possibly due to a particular conformation or change in the binding pocket or in the environment surrounding it, without chemical degradation).

Therefore, the range of pH where colour change (due to activation, protein denaturation or other phenomena) takes place would be in principle modulated by choosing an appropriate OCP (or OCP-related) carotenoprotein. Finally, the nature of the mesoporous matrix (pore size, functionalisation of surfaces) can also have an important effect, as already demonstrated in the response to thermal treatment.

Two further points deserve to be mentioned: first, thanks to its modular NTD–CTD structure, engineering of the OCP is also possible [14]. The colorimetric response can also be modulated by combining different NTDs and CTDs into artificial OCPs.

In addition, OCP has already been coupled to fluorescent systems using different approaches [11,12,32,42], and it has been shown that OCP photoactivation modulates the fluorescence response. In this paper, we show that pH-induced OCP activation can also quench the emission of fluorophores.

These results therefore also open the road to new possible research directions, for instance, using photoactive OCP-based fluorescent chimeras encapsulated in mesoporous matrices or exploiting the OCP-induced quenching of fluorescence of artificial dyes co-adsorbed on the same solid support.

It should finally be mentioned that the research can be extended to other carotenoproteins; for instance, astaxanthin-binding proteins have been largely investigated as possible sensors for temperature, pH or specific molecules (see, for instance, [43,44,45]). Two interesting carotenoproteins are the microalgal AstaP (which can bind not only asthaxanthin but also three other different carotenoids [46]) and the carotenoid-binding protein from silkworm *Bombyx mori* (BmCBP), which can bind four different carotenoids [47].

## 5. Conclusions

In the present work, activation and denaturation—by pH and by temperature—of two different OCPs (containing ECN or CAN) were investigated in mesoporous silica SBA-15 nanoparticles. The OCP colorimetric response was found to be very sensitive to pH changes in the acidic range, but interestingly this response was dramatically different from to the one observed in solution, pinpointing a key role of the silica surface. In both cases (SBA-15 matrices and solutions), pH-induced colour changes are related either by orange-to-red OCP activation or by carotenoid loss (with yellow colour) from the denatured protein.

The response of OCP proteins in raw SBA-15 matrices is particularly promising. A sharp change in the Vis absorption spectrum and in colour is observed for pH changing from 2 to 3 (in the case of OCP(CAN) in SBA-15: λ_MAX_ shifts from 454 to 508 nm) and for pH changing from 3 to 4 (in the case of OCP(ECN) in SBA-15: λ_MAX_ shifts from 445 to 505 nm).

These results represent a proof of principle for the future development of OCP-based nano-sized colorimetric sensors for pH. This represents an important step because (i) samples are nano-sized and can therefore be used in microdevices or microsystems; (ii) given that SBA-15 is often used as a scaffold to immobilise enzymes [21], OCP can be used as a tool to investigate local pH changes inside the nanoparticle, a key feature given that, at acidic pH, some enzymes become inactive. The use of different OCP binding other carotenoids, of OCP mutants (see, for instance, [48] where some activated mutant OCPs have a strong redshift compared to wild-type OCP) and a more subtle tuning of internal pore diameters are likely to lead to improved performance. Surface functionalisation is also another possible strategy (experiments are under way in our laboratories).

The effect of temperature on spectral responses of OCPs in SBA-15 matrices also gave interesting results. In this case, the study was extended to eight different kinds of SBA-15 nanoparticles (with different pore sizes and with the silica surface functionalised by aminopropyl groups). The temperature response was found to be highly dependent on the properties of the particular mesoporous silica matrix used, and in at least two cases, a three-colour temperature response was observed in the 25–60 °C range. Again, these results represent a proof of principle for the future development of OCP-based nano-sized colorimetric sensors for temperature.

Finally, we have also shown that simultaneous encapsulation in selected surface-functionalised SBA-15 nanoparticles of appropriate fluorophores makes it possible to develop OCP-based pH-sensitive fluorescent nanosystems. Fluorescent sensors are in general more sensitive than colorimetric ones; furthermore, they are better suited for detection of local pH changes at the nm scale.

In summary, the present results therefore represent a proof of principle on the possibility of exploiting OCP proteins in SBA-15 matrices as colorimetric and fluorimetric sensors for pH and temperature. Current efforts in our laboratories are aimed at improving their performance and extending the study to other carotenoproteins.

We would like to add that the present results might also help us better understand the orange-to-red activation process in OCP, which is still a matter of intense debate; in particular, so far little attention has been paid to the non-light-induced activation pathways. It is important to highlight that we showed that, in pH or temperature-induced activation of OCP, the nature of the carotenoid (ECN vs. CAN) plays a key role. It is possible that the detailed photoactivation mechanism of OCP depends on the carotenoid acting as chromophore. Experiments are currently under way in our laboratory to clarify this issue.

## Figures and Tables

**Table 1:** Comparison of different pH sensors based on protein encapsulated in mesoporous silica. As it can be observed, the OCP-based sensing nanodevices developed in this work cover the range of acidic pH levels differently from the other nanodevices, which are more adapted for a response to pH changes at around pH 7.

Sensing Element	Sensing Methodology	pH Range	Mesoporous Silica Support	Reference
Yellow fluorescent protein-based sensor	ratiometric/fluorimetric sensor	pH 6.5–7.5 or pH 7.0–8.0 according to the ratio used	Trisoperl controlled pore glass beads pore size 161.2 nm	[26]
SNARF1-labelled bovine serum albumin	ratiometric/fluorimetric sensor	pH 6–8	SBA-15, average pore size 7.8 nm	[40]
SNARF1-labelled Feruloyl esterase	ratiometric/fluorimetric sensor	pH 6–8	SBA-15, average pore size 7.8 nm	[40]
mTurquoise2 and mNeonGreen FRET-based nanodevice	ratiometric/fluorimetric sensor and FRET	pH 5.5–8.0	MSN average pore size 100 nm	[41]
OCP(echinenone)-based sensor	Colorimetric	pH 3–4	SBA-15, average pore size 8 nm	This work
OCP(canthaxanthin)-based sensor-	Colorimetric	pH 2–3	SBA-15, average pore size 8 nm	This work
OCP(canthaxanthin) + Cy3 + Cy5-based sensor	Fluorescence quenching	pH 1–2	SBA-15 with internal surfaces functionalised by aminopropyl groups; average pore size 6 nm	This work

## Data Availability

Experimental data can be provided upon request.

## Supplementary Materials

The following are available online at https://www.mdpi.com/article/10.3390/mi14101871/s1, Figure S1: SEM/TEM images of SBA-15-120 nanoparticles; Figure S2: Vis spectra of OCP(CAN) in SBA-15-80-NH_2_ in the pH 1–7 range; Figure S3: Vis spectra of OCP(ECN) in SBA-15-80-NH_2_ in the pH 1–7 range.
