# Supplementary material for: Orange Carotenoid Protein in Mesoporous Silica: A New System towards the Development of Colorimetric and Fluorescent Sensors for pH and Temperature
Source: Micromachines (Basel). 2023 Sep 29;14(10):1871. doi: 10.3390/mi14101871 (PMC10609006; doi:10.3390/mi14101871)
Supplement: Supplementary file 1 [file micromachines-14-01871-s001.zip › micromachines-2574392-supplementary.pdf]

Supplementary Information

Orange Carotenoid Protein in Mesoporous Silica: A New System towards the Development of Colorimetric and Fluorescent Sensors for pH and Temperature

Silvia Leccese, Andrea Calcinoni, Adjélé Wilson, Diana Kirilovsky, Donatella Carbonera, Thomas Onfroy, Claude Jolivalt and Alberto Mezzetti

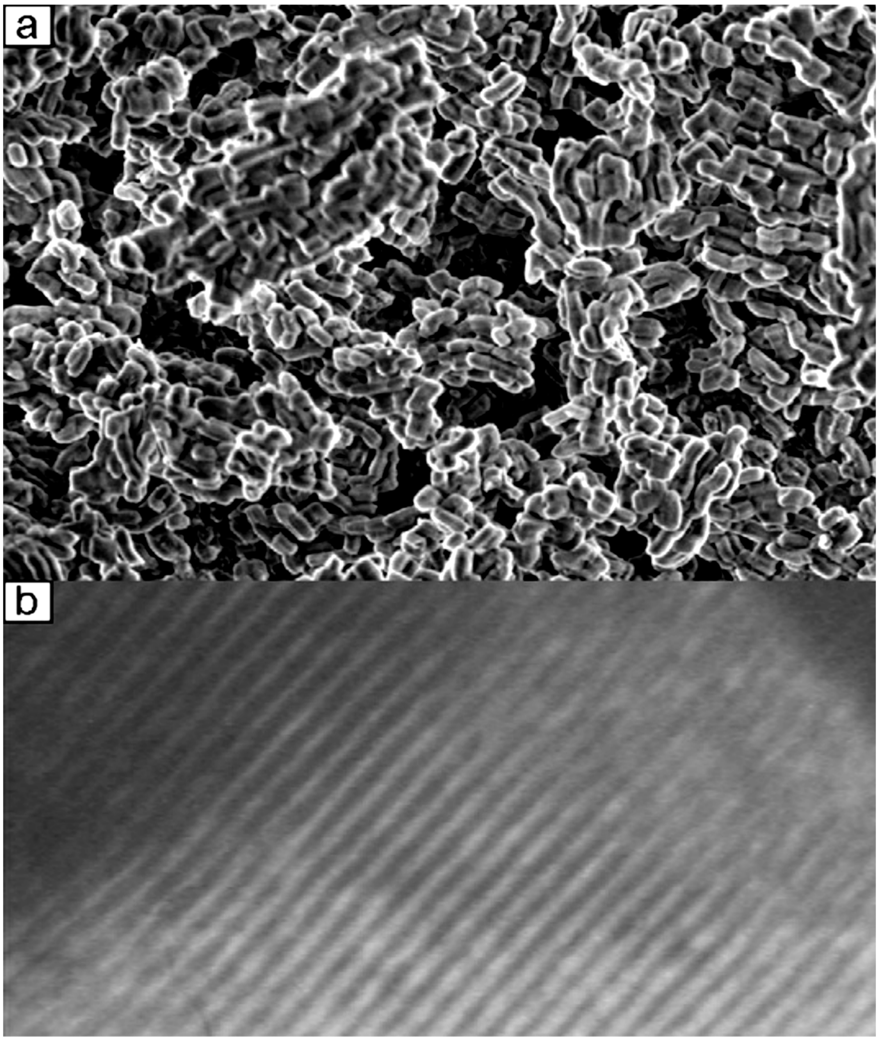

Figure S1. Morphological and structural characterization of mesoporous silica (SBA-15). (**a**) SEM image of the rod-like particles. (**b**) TEM image of the internal hexagonal mesostructured.

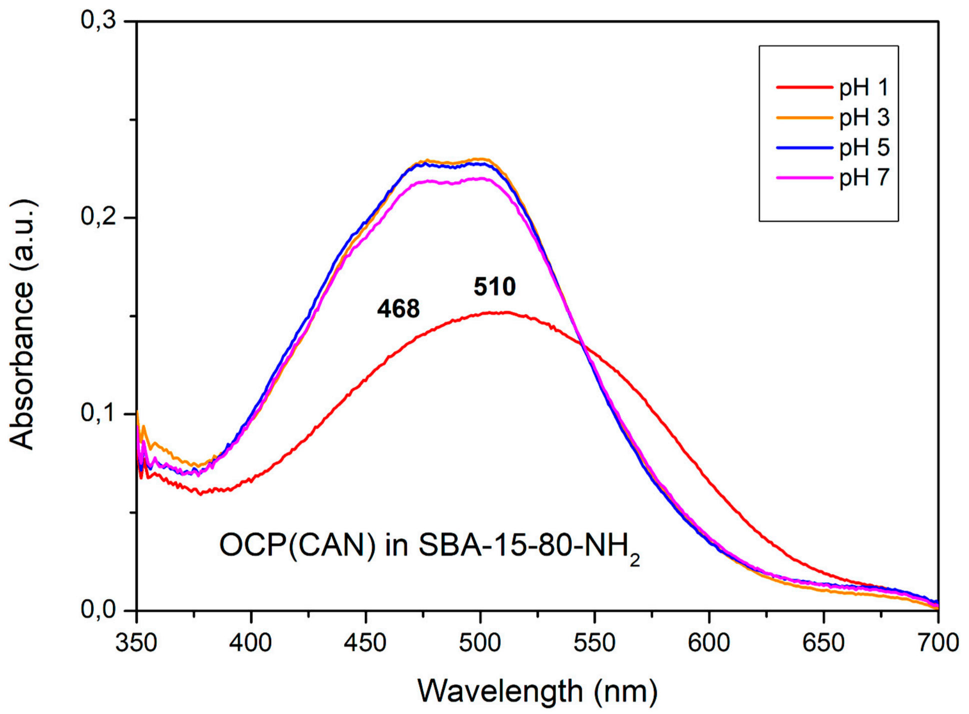

Figure S2. Vis spectra of OCP(CAN) in SBA-15-80-NH_2 in the pH 1-7 range. Experiments were carried out as follows: 10 mg of OCP-loaded silica nanoparticles were added to 1 mL of solution at a given pH. The obtained suspension was stirred for 30 minutes. After centrifugation, the obtained pellet was analysed by UV-Vis spectroscopy.

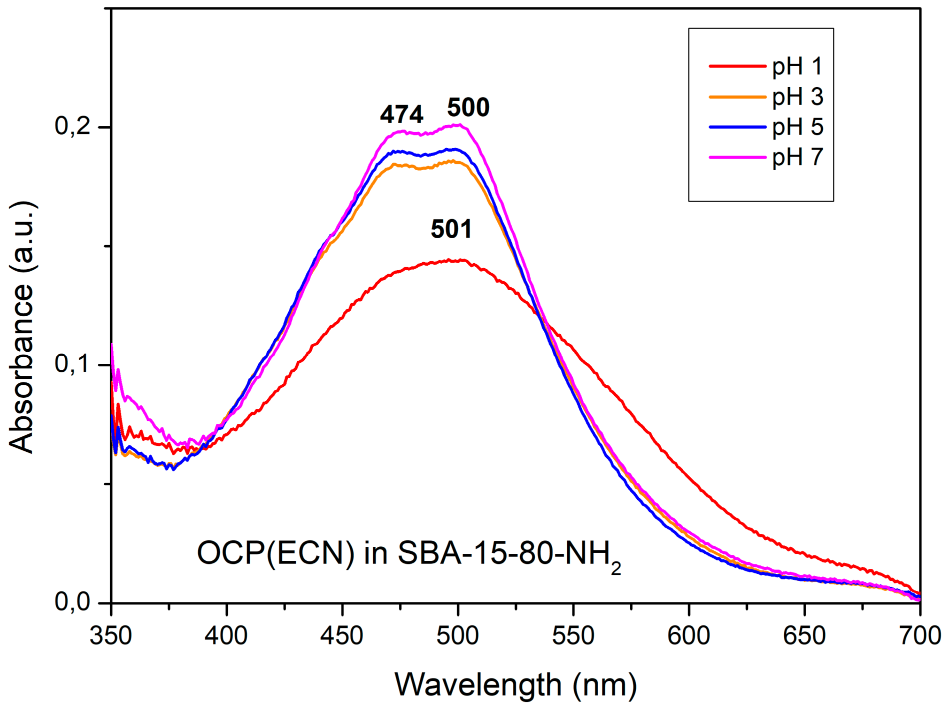

Figure S3. Vis spectra of OCP(ECN) in SBA-15-80-NH_2 in the pH 1-7 range. Experiments were carried out as follows: 10 mg of OCP-loaded silica nanoparticles were added to 1 mL of solution at a given pH. The obtained suspension was stirred for 30 minutes. After centrifugation, the obtained pellet was analysed by UV-Vis spectroscopy.
